# IQ·SPECT technology and its clinical applications using multicenter normal databases

**DOI:** 10.1007/s12149-017-1210-3

**Published:** 2017-09-22

**Authors:** Kenichi Nakajima, Koichi Okuda, Mitsuru Momose, Shinro Matsuo, Chisato Kondo, Masayoshi Sarai, Takayuki Shibutani, Masahisa Onoguchi, Takeshi Shimizu, A. Hans Vija

**Affiliations:** 1 Department of Nuclear Medicine, Kanazawa University, 13-1 Takara-machi, Kanazawa, 920-8641 Japan; 2 Department of Physics, Kanazawa Medical University, Uchinada, Kahoku, Japan; 3 Department of Diagnostic Imaging and Nuclear Medicine, Tokyo Woman’s Medical University, Tokyo, Japan; 4 Koishikawa Yanagimachi Clinic, Tokyo, Japan; 5 Department of Cardiology, Fujita Health University Hospital, Toyoake, Japan; 6 Department of Quantum Medical Technology, Institute of Medical, Pharmaceutical and Health Sciences, Kanazawa University, Kanazawa, Japan; 7 Siemens Healthcare, K. K., Tokyo, Japan; 8 Siemens Medical Solution USA, Inc., Molecular Imaging, Hoffman Estates, IL USA

**Keywords:** Myocardial perfusion imaging, Multifocal collimator, Attenuation correction, Prone imaging, Japanese Society of Nuclear Medicine (JSNM) normal database, Short-time acquisition

## Abstract

IQ·SPECT (Siemens Medical Solutions) is a solution for high-sensitivity and short-time acquisition imaging of the heart for a variable angle general purpose gamma camera. It consists of a multi-focal collimator, a cardio-centric orbit and advanced iterative reconstruction, modeling the image formation physics accurately. The multi-focal collimator enables distance-dependent enlargement of the center region while avoiding truncation at the edges. With the specified configuration and a cardio-centric orbit it can obtain a fourfold sensitivity increase for the heart at the center of the scan orbit. Since IQ·SPECT shows characteristic distribution patterns in the myocardium, appropriate acquisition and processing conditions are required, and normal databases are convenient for quantification of both normal and abnormal perfusion images. The use of prone imaging can be a good option when X-ray computed tomography (CT) is not available for attenuation correction. CT-based attenuation correction changes count distribution significantly in the inferior wall and around the apex, hence image interpretation training and additional use of normal databases are recommended. Recent reports regarding its technology, Japanese Society of Nuclear Medicine working group activities, and clinical studies using ^201^Tl and ^99m^Tc-perfusion tracers in Japan are summarized.

## Introduction

Nuclear cardiology is one of the major fields of nuclear imaging. At present, approximately 9 million myocardial perfusion imaging (MPI) studies are performed annually in the United States and 250 thousand studies in Japan including perfusion, fatty acid imaging and sympathetic nerve imaging [1]. Most of the MPI studies are performed with stress and rest with electrocardiographic gating of 8–16 frames per cardiac cycle. In the conventional single-photon emission computed tomography (SPECT) studies, the acquisition time is 15–30 min for each stress and rest study, and the total administration of ^99m^Tc-methoxyisobutylisonitrile (MIBI) and tetrofosmin is high, at least 740–1110 MBq per study [2]. Therefore, according to the necessity of short-time acquisition and reduction of radiation burden, novel ideas for cardiac studies have been sought. The first method, IQ·SPECT (Siemens Medical Solutions USA, Inc., Hoffman Estates, IL, USA) uses a multifocal collimator called SMARTZOOM for heart-focused collimation to achieve magnification of the heart without truncation of the torso, together with cardio-centric acquisition and a dedicated reconstruction method. The increased sensitivity in IQ·SPECT acquisition allows for one-fourth or even one-eighth the time gated SPECT data acquisition [3, 4]. The reconstruction allows for CT-based attenuation correction (AC), energy window-based scatter correction (SC), and it always includes distance-dependent resolution recovery. The details are described elsewhere [5]. Alternatively, cadmium–zinc–telluride (CZT) based scintillation systems use a multi-pinhole or swiveling parallel-hole collimators for preferential scanning of the heart. While MPI using CZT systems is shown to have favorable diagnostic sensitivity for angiographically-significant CAD, whether these systems confer increased diagnostic specificity over MPI with Anger-SPECT still remains elusive [6].

With respect to radiopharmaceuticals used for MPI, ^201^Tl is still widely used in Japan. Approximately half of the MPI studies use ^201^Tl, whereas the other half uses ^99m^Tc labeled myocardial perfusion tracers [7, 8]. Moreover, use of ^123^I labeled fatty acid and sympathetic nerve imaging studies acquired with 360° and 180° camera rotations are also common in Japan. Considering these specific situations in Japan, normal databases for quantification fitted for tracers and acquisition methods are indispensable [9, 10].

This review focuses on IQ·SPECT technology, and specific normal databases created by multicenter collaboration and its effective use in clinical studies. In particular, due to its unique collimation, some cautions are required for data acquisition, image reconstruction and image interpretations. Some tips for using this new technology will also be discussed [11]. We further include basic characteristics of ^201^Tl imaging and clinical application using IQ·SPECT system.

## Characteristics of IQ·SPECT technology

**Fig. 1:**
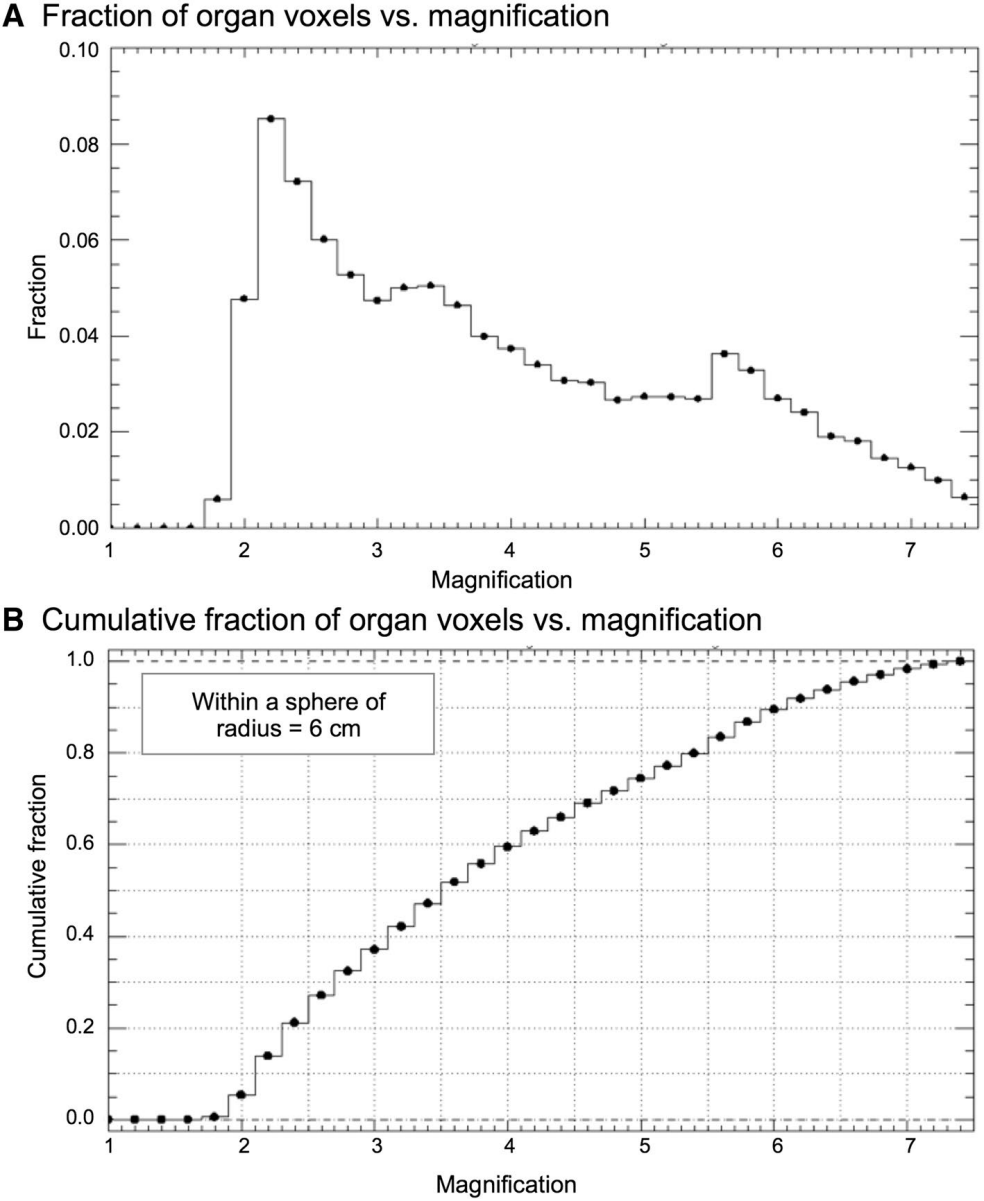
Fraction of organ voxels versus magnification (**a**) and cumulative fraction (**b**). The computation assumes a sphere with a 6 cm radius within the acquisition orbit of 28 cm radius

IQ·SPECT consists of three unique components: a SMARTZOOM collimator, rotational orbit around the heart, and ordered subset conjugate gradient minimizer (OSCGM) methods that enable an effective short-time acquisition and reconstruction. The collimator provides specific geometry to magnify a region around the heart, enabling fourfold higher sensitivity than conventional MPI with a low-energy high-resolution (LEHR) collimator (hereinafter referred to as conventional MPI) [4]. The holes are focused at the heart around the center of the collimator and are near-parallel at the edge avoiding truncation of the torso following a design equation. To understand the magnification characteristics, it is instructive to compute the angle averaged magnification for the 208° orbit at 28 cm which is a representation of the tomographic magnification. If one assumes a sphere with a radius of 6 cm at the center of the acquisition orbit with a 28 cm radius, then about 80% of all voxels have a magnification *m* ≥ 2, and 40% of all voxels have a *m* ≥ 4 and 10% have *m* ≥ 6, as can be seen in Fig. 1. Figure 1a shows the histogram of the voxels with a bin size of magnification Δ*m* = 0.2 from 1 to 7.5, and Fig. 1b shows the cumulative distribution, namely, the integral of the curve in Fig. 1a. The sweet spot is comprised of voxels with *m* ≥ 1, but in practice it is the volume centered at 28 cm encompassing the heart where more than 80% of voxels have an *m* ≥ 2, as illustrated in a sphere with a 6 cm radius, representing a volume of about 0.9 L with an average magnification of 4.04, a standard deviation of 1.48, with maximum and minimum magnification of *m* = 7.59 and *m* = 1.9, respectively.

The imaging trade-off is between high magnification and low resolution, and in this application the objective is to improve imaging efficiency without degradation of resolution as compared to LEHR imaging using the FBP reconstruction with patient contouring orbit. The acquisition orbit is centered at the heart (cardio-centric orbit) to maintain the myocardium inside of the volume with an average magnification of *m* ≈ 4 during the data acquisition, at the design distance of 28 cm and using a symmetric 208° orbit. Due to its focusing geometry, the photon path lengths between neighboring emitting voxels are slightly different compared to LEHR image formation, and correspondingly the attenuation induced discrepancy is more emphasized, which in effect creates the impression of a “hotter” apex. Attenuation artifacts are in principal similar to those of LEHR imaging, yet they have a characteristic attenuation pattern due to the more complex imaging geometry. When we simply observe the IQ·SPECT short-axis and long-axis images, the attenuation artifacts may cause difficulty in image interpretation, particularly in the inferolateral area. These kinds of artifacts can be significantly reduced by appropriate CTAC [12]. In addition, the prone imaging may be more comparable to the conventional MPI as discussed later [13].

**Fig. 2:**
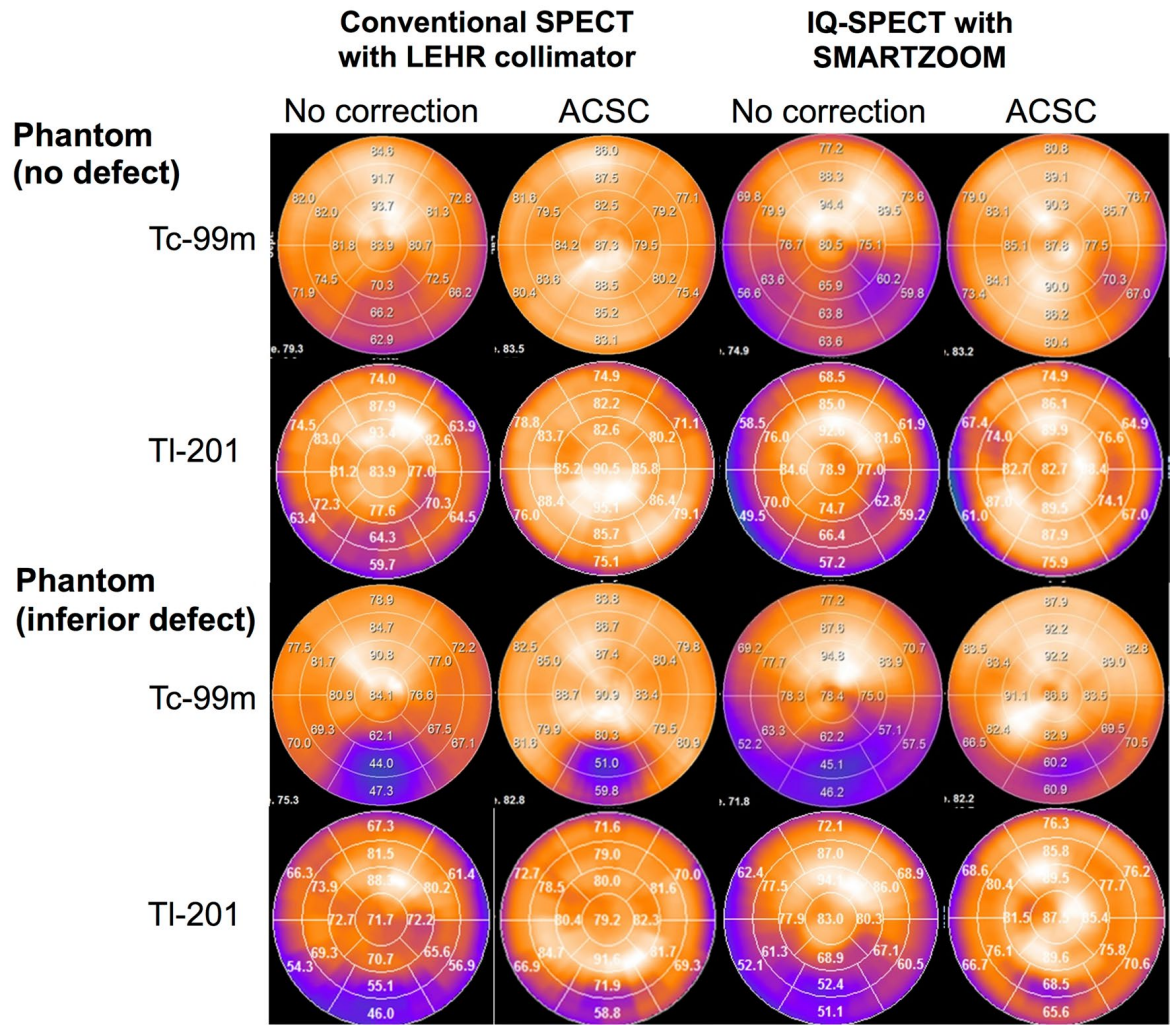
Phantom experiments with and without an inferior defect. The conventional SPECT images with LEHR collimator and SMARTZOOM collimator are compared with and without attenuation and scatter corrections (ACSC)

To better understand the characteristics of IQ·SPECT imaging compared to the conventional MPI, the following phantom experiments are conducted in the Kanazawa University Hospital (TS and MO). Figure 2 shows the polar maps of cardiac phantoms in torso with no correction (NC) and with attenuation and scatter corrections (ACSC) using ^99m^Tc and ^201^Tl in a normal and defective myocardium. The defective part of myocardial phantom was simulated with a rectangular acrylic sector (30 × 20 mm) filled with non-radioactive water to mimic transmural infarct that was placed in the mid-to-basal inferior wall of the left ventricle.

The NC image of IQ·SPECT showed reduced counts in the inferolateral wall of normal myocardium, which was not observed in the conventional MPI. When ACSC was used, the false defective area in the inferolateral wall disappeared, and count distribution was similar to that of the conventional MPI. A similar tendency was observed in both ^99m^Tc and ^201^Tl images. In a phantom with the inferior defect, although the %uptake in the NC image with IQ·SPECT was similar to that of the conventional MPI, %uptake of the inferior walls in the ACSC image was approximately 5–10% higher than that acquired with the conventional MPI. In addition, the %uptake of the inferior walls in ^99m^Tc images was lower than that of ^201^Tl for both the conventional MPI and IQ·SPECT.

Further technological studies showed first that the OSCGM algorithm had different characteristics compared to ordered subset expectation maximization (OSEM) reconstruction. In the OSEM method, similar image quality was obtained when a product of subsets and iterations (updates) was the same. However, in the OSCGM method, even the same update number may create different distribution as the number of projection views is limited [14]. The optimal update recommended from our study was approximately 30 with a subset of 1 and iterations of 30 for gated SPECT data and a subset of 3 and iterations of 10 for non-gated SPECT data. Reconstruction with more than 3 subsets is not recommended, as there are only 34 views.

Second, the ^201^Tl image is generally acquired using either a single-energy (SE) window setting with a characteristic X-ray peak of 69–83 keV or a dual-energy (DE) window setting with additional γ-rays of 167 keV. The non-corrected DE image was useful for reducing the attenuation artifact of the inferoseptal and inferior walls for NC, which is sometimes difficult to differentiate from the true defect. Since the defect detectability of DE image with AC was shown to be significantly decreased, the combination of the SE window setting and ACSC may be recommended for the optimized image quality [15].

Third, while a small heart causes reduced end-diastolic volume and high ejection fraction [16], the accuracy of left ventricular function with IQ·SPECT did not improve regardless of the magnified image acquisition. The left ventricular function of IQ·SPECT with OSCGM method showed the same tendency as the filtered back projection method [17].

The initial consideration of optimized clinical acquisition and reconstruction protocols aligned with the manufacturer’s recommended acquisition and processing conditions. These conditions are summarized in Table 1.

**Table 1:** Recommended acquisition protocol for IQ·SPECT

Acquisition parameters
Pixel size: 4.8 mm
Matrix size: 128 × 128
Zoom: 1.00
Rotation range: 208
Number of projections: 34 (17 views/detector)
Rotation radius: 28 cm
^99m^Tc energy window:
140 keV ± 7.5%, lower scatter 15% (preset condition)
^201^Tl energy window:
70 keV ± 10%, lower scatter 20%, upper scatter 20% (preset condition)
Acquisition time:
Low dose (< 370 MBq for ^99m^Tc or any injected dose of ^201^Tl)
Dwell time: 14 s
Reconstruction parameters
Ordered subset conjugate gradient minimizer (OSCGM) method
Subset and iteration: 3 subsets, 10 iterations
Gaussian filter (full-width at half maximum) 10 mm
Corrections for ^99m^Tc: both AC and SC
Corrections for ^201^Tl:both AC and SC, or AC only

*AC* attenuation correction, *SC* scatter correction

## ^201^Tl normal database

Quantitative analysis using normal databases is helpful for interpretation of IQ·SPECT images [18]. The characteristics of ^201^Tl normal databases for supine and prone IQ·SPECT imaging and diagnostic performance of these databases for myocardial perfusion evaluation are described in this section [18, 19].

The Japanese Society of Nuclear Medicine (JSNM) working group reported on databases generated for ^201^Tl IQ·SPECT imaging. These databases consisted of 159 patients with a low likelihood of coronary artery disease (CAD), collected from four hospitals in Japan [10]. The patients underwent ^201^Tl myocardial perfusion SPECT in either supine or prone position using the IQ·SPECT system. The acquisition time per view ranged from 14 to 30 s. For the evaluation of normal databases, the mean and deviation polar maps were generated using the QPS (Cedars Sinai Medical Center, Los Angeles, USA) software.

**Fig. 3:**
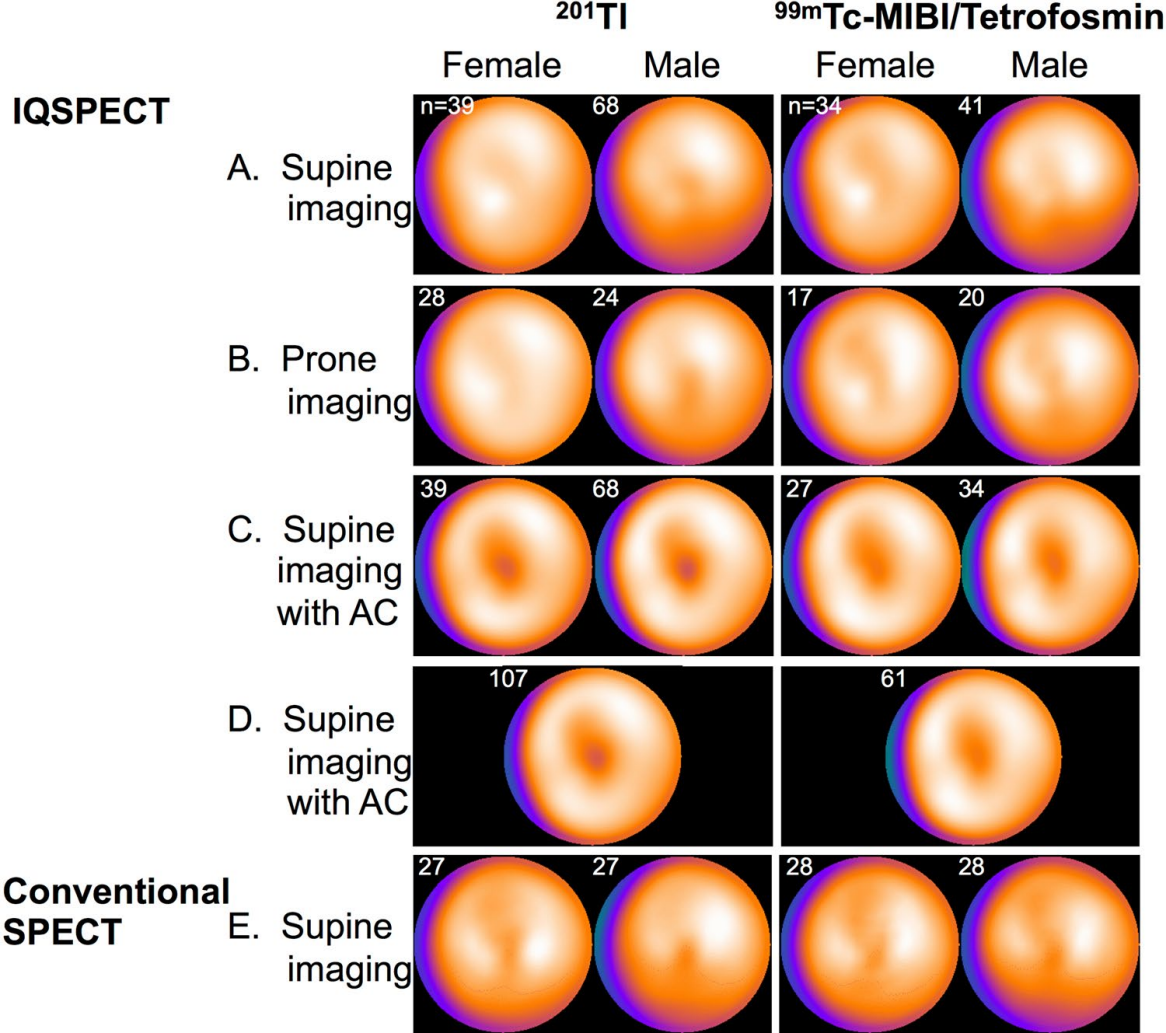
Polar maps of stress myocardial perfusion studies with ^201^Tl (left panels) and ^99m^Tc-labeled tracers (right panels) with IQ·SPECT and conventional SPECT systems. Polar maps of the male and female normal myocardial perfusion SPECT databases derived from supine IQ·SPECT images (**a**), prone IQ·SPECT images (**b**), supine IQ·SPECT images with AC (**c**), and the sex-combined normal MPI database derived from supine IQ·SPECT images with AC (**d**) were represented. The previously generated normal databases by the Japanese Society of Nuclear Medicine working group without attenuation correction were used as the reference of the conventional SPECT data (**e**). The numbers of patients comprised the normal databases that were denoted in the panels

Distributions of myocardial perfusion varied among uncorrected supine normal databases, uncorrected prone normal databases, and supine normal databases with AC as well as conventional MPI normal databases (Fig. 3). The mean myocardial segmental counts observed at the inferior and inferolateral walls in the supine normal databases were lower compared to those in the prone normal databases. Reduced myocardial counts at the apical regions (apical thinning) were also observed in the images acquired in the supine position with AC. For AC databases, sexes were combined because there was only borderline significance seen in the apical region between sexes. In addition, the automatic scoring of summed stress score (SSS) with combined database still resulted in high agreement with the results from sex-segregated database [19].

According to the retrospective validation study in which patients underwent double scans of IQ·SPECT and the conventional MPI, the diagnostic performance of IQ·SPECT using ^201^Tl normal databases was comparable with that of the conventional MPI [18, 19]. A total of 36 patients underwent ^201^Tl adenosine stress–rest MPI. The acquisition time of the IQ·SPECT system was approximately one-quarter of the standard duration of the conventional MPI. The SSS was compared using the acquisition condition-matched male and female normal databases. There were no significant differences in SSS, sensitivity, specificity, and accuracy for detecting CAD between the conventional MPI and IQ·SPECT with AC. The area under the curve by the receiver-operating characteristic analysis was also comparable.

## Normal database for ^99m^Tc-labeled myocardial perfusion tracers

In addition to the previously reported ^201^Tl normal databases, we have also generated male, female and sex-combined IQ·SPECT normal databases for ^99m^Tc-MIBI and tetrofosmin imaging. The ^99m^Tc IQ·SPECT normal databases consisted of 112 patients with a low likelihood of CAD collected from three hospitals in Japan. The patients underwent ^99m^Tc-MIBI and tetrofosmin MPI in either supine or prone position using the IQ·SPECT system. The acquisition time per view ranged from 9 to 30 s.

The characteristics of myocardial perfusion distribution of ^99m^Tc normal database are shown in Fig. 3. Low myocardial counts were observed in the inferior and inferolateral walls in the supine position. Prone imaging compensated attenuated inferior myocardial counts to some extent. The inferior myocardial counts showed lower values in males than in females. Although AC compensated for inferior low myocardial counts, low counts at the apex were observed. As in the case of ^201^Tl, since AC provided similar myocardial perfusion distributions in male and female normal databases, a sex-combined normal database was generated.

When the myocardial perfusion distributions of IQ·SPECT ^99m^Tc and ^201^Tl normal databases were compared, myocardial perfusion distributions were visually similar irrespective of radionuclides. However, attenuation-corrected myocardial count at the apex was lower in ^201^Tl supine imaging than in ^99m^Tc supine imaging (76 vs. 81%, *p* = 0.0020). Regarding the sex difference of myocardial perfusion distribution, lower inferior myocardial distributions were observed in males than in females in both ^99m^Tc and ^201^Tl normal database for supine and prone IQ·SPECT imaging.

These JSNM working group normal databases of IQ·SPECT for both ^201^Tl and ^99m^Tc tracers are available now for clinical studies/research in addition to the conventional MPI databases (stress and rest), fatty acid and sympathetic imaging (early and late) with 180° and 360° rotations [10].

## IQ·SPECT images in normal subjects

**Fig. 4:**
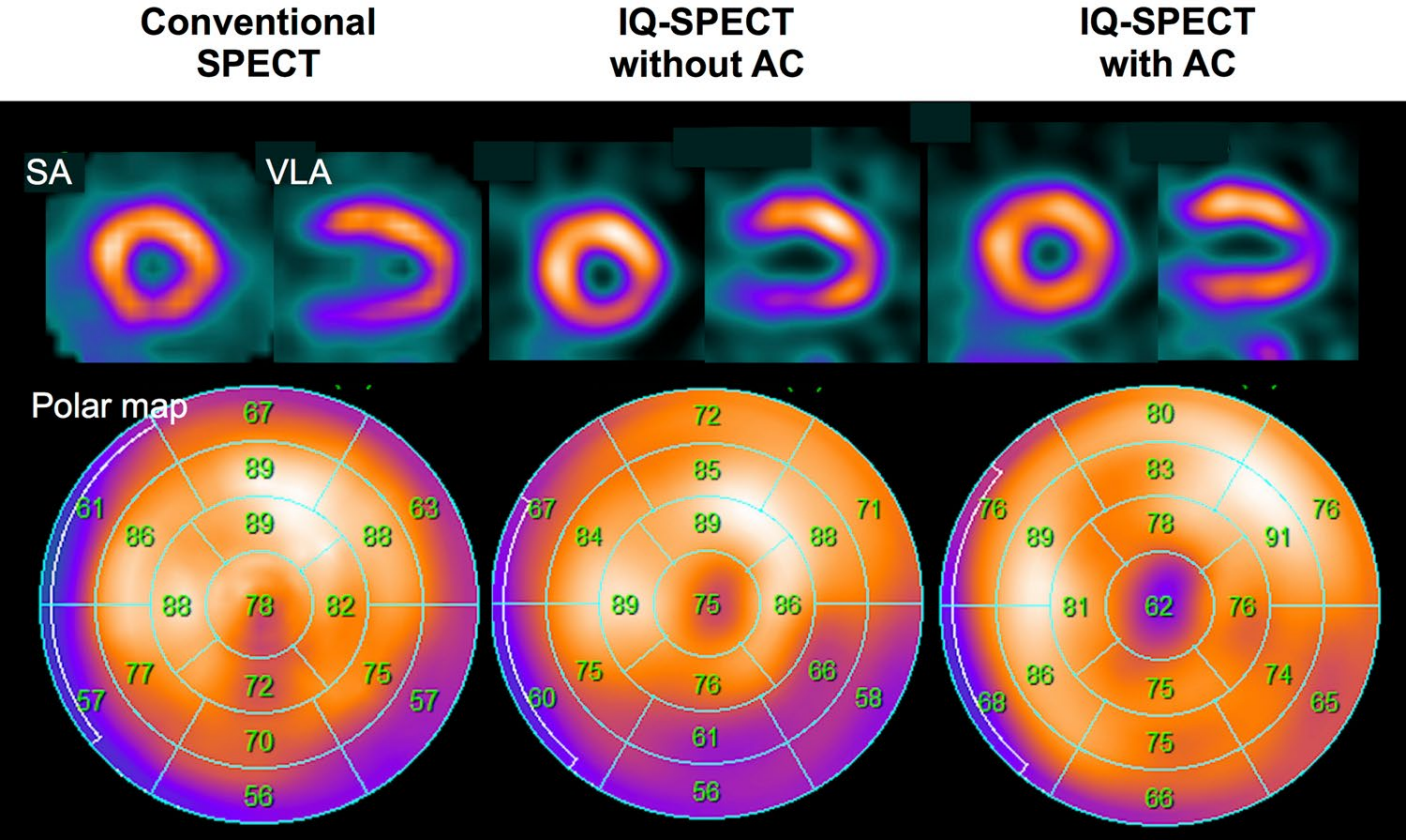
SPECT images of a normal subject with three SPECT conditions. Conventional MPI (left), IQ·SPECT without (mid) and with attenuation correction (AC) (right) are shown. *SA* short-axis, *VLA* vertical long-axis

To recognize normal subjects as definitively normal has a practical value in patients suspected of having CAD. The key point for correctly interpreting normal myocardial images obtained with IQ·SPECT is to understand the characteristic findings of the apex, apical anterior and inferolateral walls of the left ventricle [20–22]. In the CTAC images, the apical counts are frequently decreased even in normal subjects (Fig. 4). One of the causes of low activity in the apex of the left ventricle is due to the anatomically thin apex. Even with cardiac CT imaging, the apical wall thickness of the left ventricle is relatively reduced [23]. A relatively larger motion of the apex, either beating or respiratory motions, may be another reason. Therefore, the apparent decrease in apical counts with an AC image might rather reflect these phenomena correctly.

In the conventional ^201^Tl MPI study, it is challenging to determine whether the low counts in the inferior wall are due to an artifact. Although the low inferior activity is a common finding, the attenuation artifact in ^201^Tl perfusion study is more pronounced compared to that in a ^99m^Tc study.

The characteristics of normal IQ·SPECT images without CTAC are slightly different from those of conventional MPI images. Therefore, the attenuation and scatter of the photons in the patient’s tissues must be considered. Since the location of the attenuation artifact depends on the position of the soft tissues near the left ventricle, the CT attenuation map of the surrounding structures, such as liver and breasts can be useful in each patient. The IQ·SPECT with CTAC improves this attenuation effect of the inferior wall. Even in clinical patients with low likelihood of CAD, when proper AC is applied, IQ·SPECT images improve the inferior wall artifacts compared to the anterior wall [20].

While the attenuation of the inferior wall is improved with CTAC (Fig. 4), a tendency of apical thinning is frequently observed. In our experience, IQ·SPECT with CTAC is effective in correcting the false-positive defect of the inferior wall in both ^201^Tl and ^99m^Tc studies. Needless to say, gated SPECT will help a nuclear physician diagnose normal perfusion. When normal wall motion and thickening are observed on the gated images, judgment of normal perfusion could be supported. It is also desirable to utilize the normal databases as practical quantitative criteria for judgment.

## Clinical characteristics of IQ·SPECT without attenuation correction

**Fig. 5:**
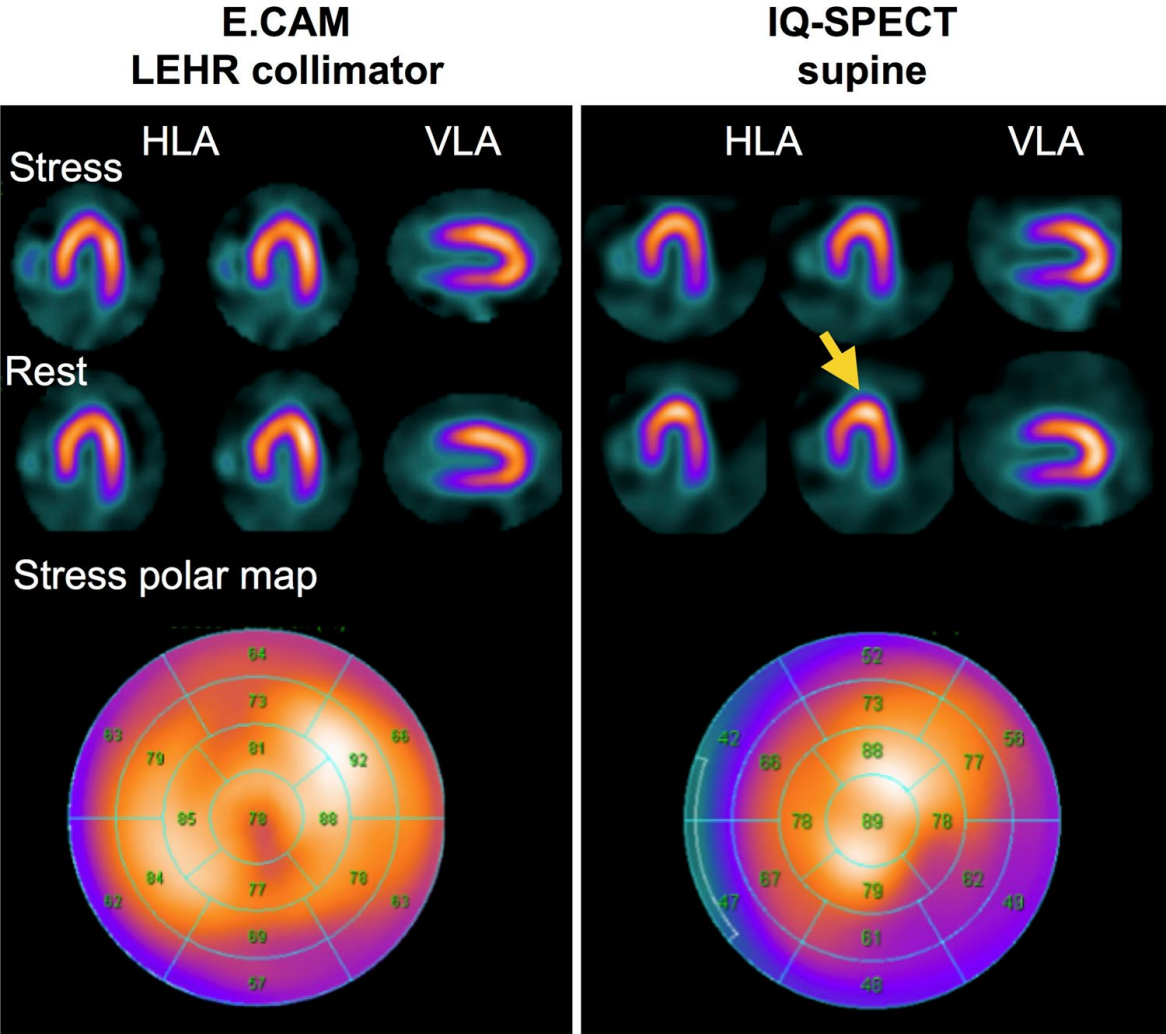
Comparison of conventional MPI (E.CAM, Siemens) in the supine position and IQ·SPECT. IQ·SPECT images and polar map show hot apex (arrow) and inferolateral attenuation. *LEHR* low-energy high-resolution, *HLA* horizontal long-axis, *VLA* vertical long-axis

For IQ·SPECT images without AC, characteristic artifacts are often observed. The hot apex phenomenon as discussed above appears in IQ·SPECT as compared with the conventional MPI. In Fig. 5, two kinds of SPECT images are shown from a patient who underwent double-scans of supine conventional MPI and IQ·SPECT under exercise-induced stress. Therein, IQ·SPECT image represents the characteristic attenuation patterns despite almost all normal distributions with slight posterior attenuation on the conventional MPI. This figure highlights the necessity of understanding the physical characteristics of attenuation effects on IQ·SPECT images when interpreting without corrections in the image reconstruction. The following studies were conducted at Tokyo Woman’s Medical University (KC, MM) [24].

**Fig. 6:**
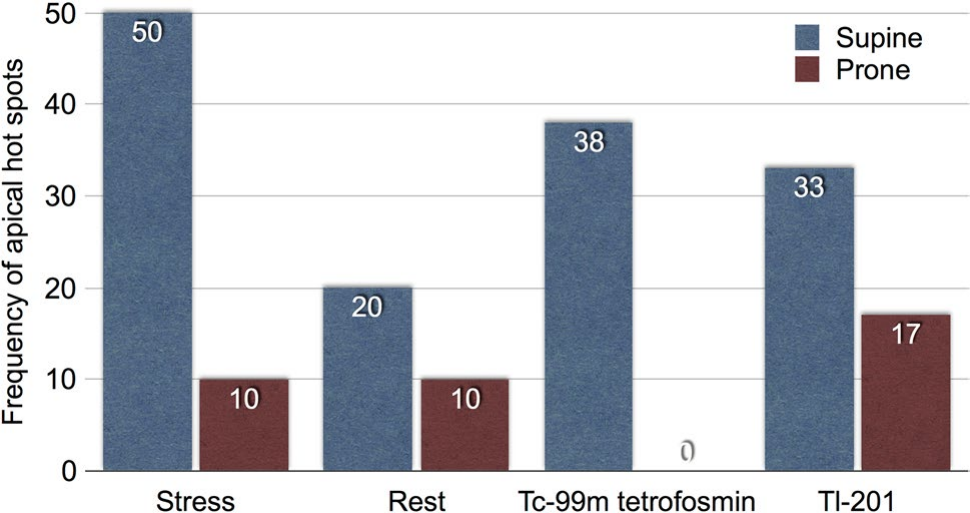
Frequency of the hot apex in the supine and prone positions

We assessed tracer distribution in normal patients acquired by IQ·SPECT and the conventional MPI to clarify respective imaging characteristics. Ten patients including 3 females who were judged normal in the stress MPI and other clinical correlates were evaluated. Six patients were scanned with ^201^Tl, while four patients were scanned with ^99m^Tc tetrofosmin, respectively. Each patient underwent IQ·SPECT acquisition in both supine and prone positions following the conventional MPI in the supine position. Attenuation and scatter corrections were not applied to these cases. We assessed the frequency of the hot apex in the IQ·SPECT supine and prone images (Fig. 6). The results indicated that the hot apex occurred more frequently in IQ·SPECT supine than in prone images: 7 of 20 (35%) vs. 2 of 20 (10%).

**Fig. 7:**
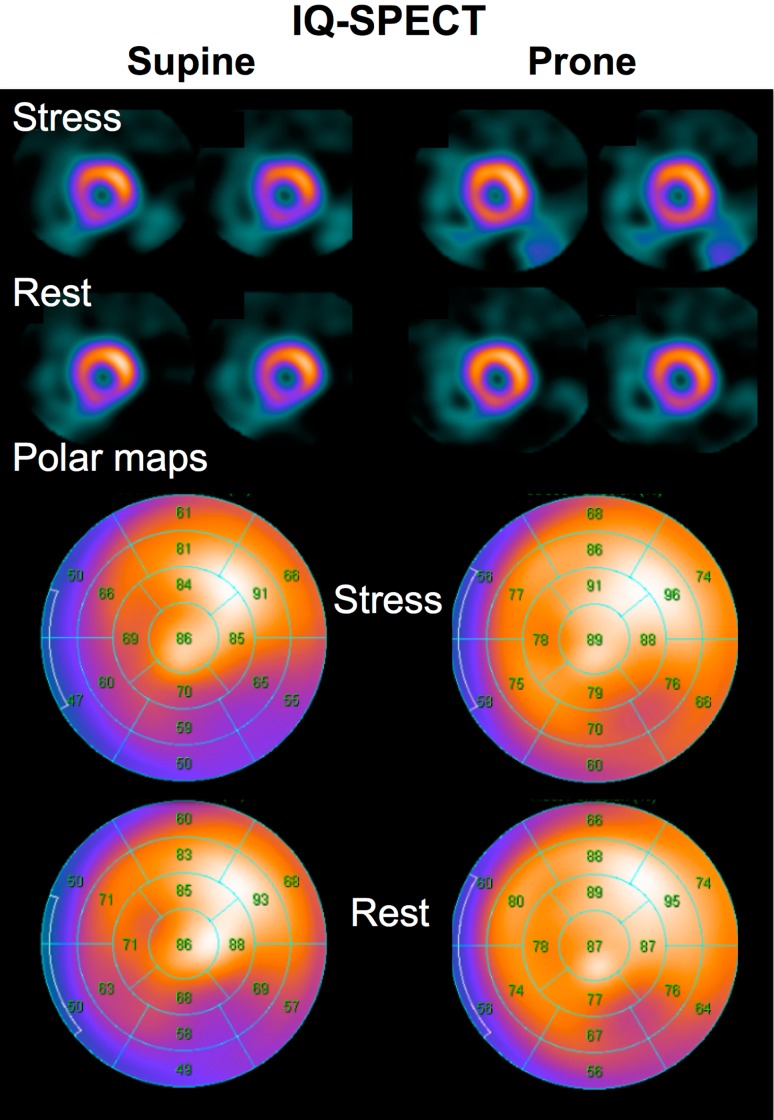
Comparison of IQ·SPECT images in the supine and prone positions. The prone image improves low counts in the inferior wall that is observed in the supine position

Semi-quantitative assessment of myocardial %uptake supports the visual analysis. The IQ·SPECT images with supine and prone positions showed increased %uptake in anterior to apical areas compared to the conventional MPI. Supine IQ·SPECT images without corrections showed lower %uptake in the septal and inferolateral regions than the conventional MPI. IQ·SPECT prone positioning improved the inferolateral attenuation seen in the supine IQ·SPECT (Fig. 7). Overall, IQ·SPECT images in the prone position are mostly homogeneously distributed in normal patients among the 3 acquisition protocols.

The improvement of attenuation in the prone position may be explained as follows: the prone position makes a heart fall forward, then the heart is slightly separated from liver and diaphragm, and is slightly closer to the chest wall. Diaphragmatic attenuation may be consequently reduced [25].

Consistency between IQ·SPECT and attenuation-corrected SPECT was also examined. One hundred and sixteen consecutive patients suspected of having ischemic heart diseases underwent stress MPI with ^99m^Tc-tetrofosmin and were prospectively analyzed [24]. SPECT-CT acquisition was first performed followed by supine and prone IQ·SPECT. Twenty-six patients (22%) showed myocardial ischemia. Based on the SPECT-CT findings as a gold standard, false-positive and false-negative rates of IQ·SPECT with supine and prone imaging were evaluated in each coronary territory (Table 2). The overall false-positive and -negative rates (FR) in IQ·SPECT prone were 1.7% in all coronary territories. The overall FR in IQ·SPECT supine were 2.6% both in the left anterior descending artery (LAD) and the right coronary artery (RCA), whereas the rate was 0% in the left circumflex artery (LCX). The FR in IQ·SPECT in the prone position was slightly lower than that in the supine position, but the FR in both protocols were low, and consistency between IQ·SPECT and SPECT-CT was quite high.

**Table 2:** Inconsistency rate between IQ and SPECT-CT in each coronary territory

IQ·SPECT position	False rate	LAD	RCA	LCX
Prone	FPR	1/97 (1.0%)	2/114 (1.8%)	1/103 (1.0%)
	FNR	1/19 (5.3%)	0/2 (0%)	1/13 (7.7%)
	Overall FR	2/116 (1.7%)	2/116 (1.7%)	2/116 (1.7%)
Supine	FPR	1/97 (1.0%)	3/114 (2.6%)	0/103 (0%)
	FNR	2/19 (10.5%)	0/2 (0%)	0/13 (0%)
	Overall FR	3/116 (2.6%)	3/116 (2.6%)	0/116 (0%)

*FPR* false-positive rate, *FNR* false-negative rate, *FR* false rate, *LAD* left anterior descending artery, *RCA* right coronary artery, *LCX* left circumflex artery

**Fig. 8:**
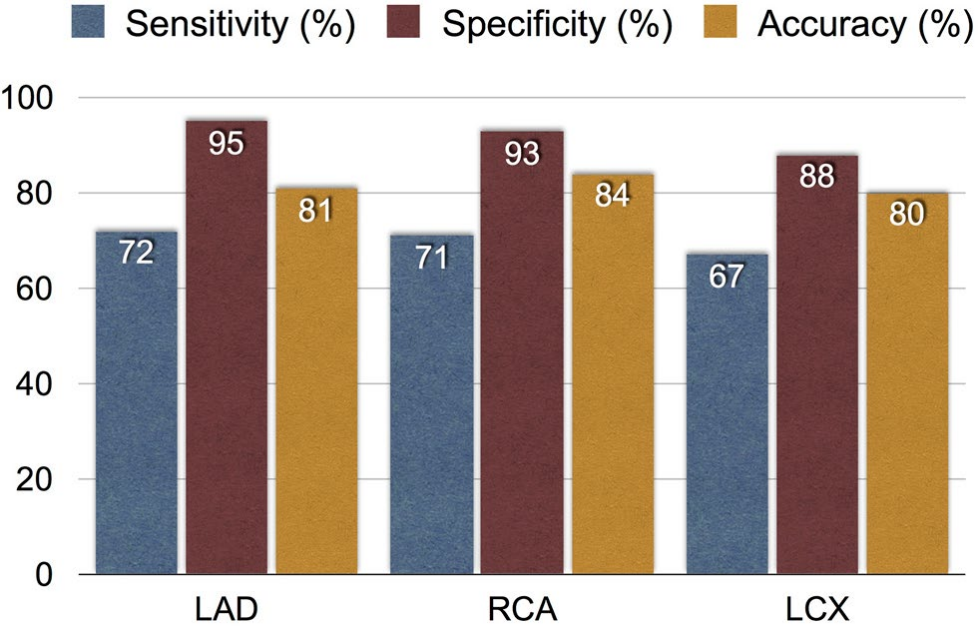
Diagnostic performance of stress myocardial perfusion imaging by IQ·SPECT using a prone position. Sensitivity, specificity and accuracy are shown in each coronary territory based on coronary angiography. *LAD* left anterior descending, *RCA* right coronary artery, *LCX* left circumflex

The diagnostic value of IQ·SPECT prone imaging was examined retrospectively in 129 consecutive patients with suspected ischemic heart disease who underwent IQ·SPECT in the prone position and coronary angiography. Significant coronary insufficiency was defined as having 75% or more stenosis, and the concordance of IQ·SPECT prone results with angiographic findings was evaluated (Fig. 8). The number of diseased coronary vessels was evenly distributed (*n* = 129; 3, 2, 1 and 0 vessel diseases, 29, 31, 34, and 35, respectively), and prevalence of diseased coronary vessels was 47%. The coronary artery-based sensitivity, specificity and accuracy were approximately 70, 90 and 80%, respectively. Specificity in the RCA territory was also approximately the same as the other coronary territories, which indicated no influence by inferior attenuation under the IQ·SPECT in the prone position.

## Clinical characteristics of IQ·SPECT with CTAC

As stated above, it is necessary to understand the attenuation physics in the inferior and the apex to apical anterior regions correctly in IQ·SPECT. Concomitantly, when CTAC is applied, the inferior counts increase, whereas the anterior and apical counts decrease.

**Fig. 9:**
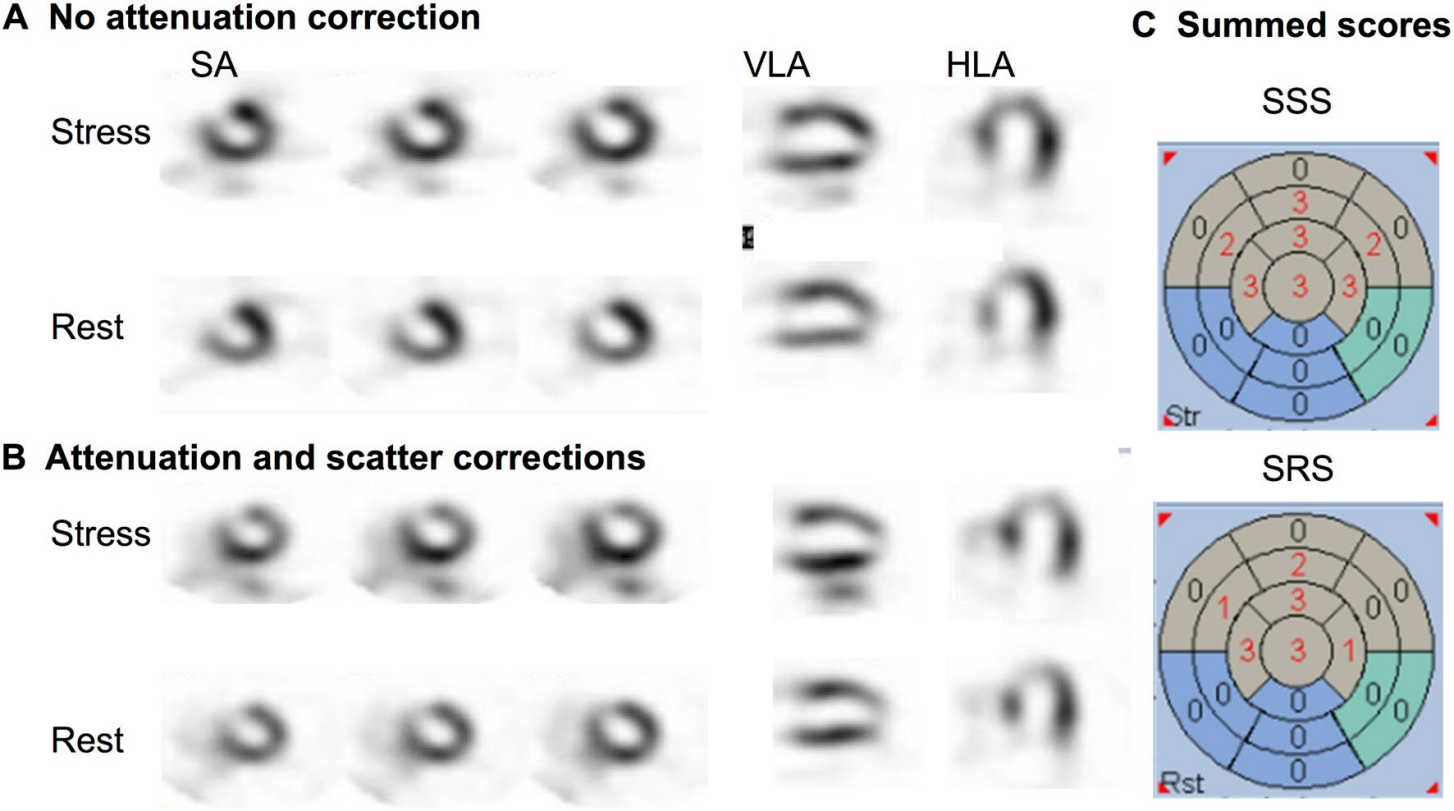
A 70-year-old man with history of myocardial infarction and percutaneous coronary angioplasty. **a** IQ·SPECT images without X-ray CT-based attenuation correction (CTAC), **b** IQ·SPECT images with CTAC, **c** quantitative scoring results with CTAC by comparison with normal databases of JSNM working group

These features are represented in Fig. 9. A patient with history of anteroseptal and apical myocardial infarction and LAD coronary artery angioplasty 9 years ago presented with recurrent atypical chest pain. Stress and rest ^201^Tl MPI was performed with IQ·SPECT. In this example, IQ·SPECT without CTAC shows characteristic anteroseptal, and apical hypoperfusion (Fig. 9a). However, CTAC images show more extensive anteroseptal and apical hypoperfusion (Fig. 9b). A slightly increased count in the inferior wall was also noticed after CTAC. These inconsistent findings may be observed between IQ·SPECT with and without CTAC. For clinically optimal judgment, it is often helpful to refer to the images without CTAC for the evaluation of the anteroseptal and apical regions, while CTAC images may be more useful for evaluating the inferior region. In particular, when findings are equivocal, it is recommended that both image sets with and without CTAC be used for the confirmation of the final judgment. In this patient, the automatic scoring of IQ·SPECT with CTAC compared against the JSNM normal databases showed consistent results with respect to the extent of anteroseptal and apical low uptake, while showing the spared inferior region (Fig. 9c).

Clinical studies performed in Japan also suggested comparable diagnostic accuracy with an IQ·SPECT short-time acquisition in patients with CAD [13, 21, 26, 27]. Although IQ·SPECT images do not show improvement in the image quality compared to the conventional MPI, either short-time acquisition or reduction of radiation exposure by low doses can be effectively achieved by IQ·SPECT without compromising the diagnostic accuracy.

## Conclusion

IQ·SPECT has a unique collimator design that enables enlargement of cardiac region without truncation. In conjunction with the cardiac centric orbit and the specific reconstruction method (OSCGM), either short-time or low dose acquisition is feasible. Since IQ·SPECT shows characteristic distribution in the myocardium in the inferolateral and apical regions, optimized acquisition and processing conditions are required. The use of prone imaging can be a good option when X-ray CT is not equipped for AC. With CTAC, since count distribution significantly changes in the inferior wall and around the apex, careful interpretation and additional use of normal databases, for example, by the JSNM working group database, are recommended.

## Abbreviations

AC: Attenuation correction
CAD: Coronary artery disease
CT: Computed tomography
CTAC: Computed tomography-based attenuation correction
LEHR: Low-energy high-resolution
MPI: Myocardial perfusion imaging
QPS: Quantitative perfusion SPECT
SC: Scatter correction
SPECT: Single-photon emission computed tomography
SSS: Summed stress score
